# Hydrophobic Radiative Cooling using Zein‐Functionalized Polyvinyl Alcohol Nanofibers with Dielectric Nanoparticles

**DOI:** 10.1002/smll.202507295

**Published:** 2025-09-24

**Authors:** Minseo Jeong, Seokgyu Kwon, Changhwan Hyeon, Juhoon Baek, Myeongsu Seong, Minkyung Kim, Dasol Lee

**Affiliations:** ^1^ Department of Biomedical Engineering Yonsei University Wonju 26493 Republic of Korea; ^2^ Department of Mechanical and Robotics Engineering Gwangju Institute of Science and Technology (GIST) Gwangju 61005 Republic of Korea; ^3^ Department of Mechatronics and Robotics School of Advanced Technology Xi'an Jiaotong‐Liverpool University Suzhou 215123 China

**Keywords:** dielectric nanoparticles, hydrophobic nanofibers, moisture‐responsive materials, radiative cooling, thermal regulation, zein‐functionalized PVA

## Abstract

Polyvinyl alcohol (PVA) is a promising material for radiative cooling owing to its high infrared emissivity and mechanical flexibility; however, its inherent hydrophilicity limits its practical applications, particularly in humid environments. In this study, a scalable and environmentally friendly approach is introduced to overcome this limitation by incorporating Zein—a hydrophobic protein derived from corn—into a PVA matrix in conjunction with aluminum oxide and silicon dioxide nanoparticles. The resulting composite nanofiber membrane, PZAS, exhibits significantly enhanced hydrophobicity, achieving an average water contact angle of 118.5° over 60 s, compared with ≈40° for pure PVA nanofibers. A high solar reflectance of 91.7% and strong infrared emissivity of 96.9% within the atmospheric transparency window are demonstrated. In outdoor measurements, a temperature reduction of up to 6.9 °C is achieved below ambient temperature. These findings underscore the potential of PZAS as a viable and sustainable radiative cooling material for applications in thermal regulation, particularly in textiles and wearable cooling technologies. The initial hydrophobicity followed by gradual water absorption of PZAS highlights its suitability for advanced biomedical applications, including moisture‐managing textiles, sweat‐based biosensors, and controlled drug delivery systems.

## Graphical abstract

A high‐performance radiative cooling material is fabricated from hydrophilic polyvinyl alcohol using a green, scalable method. The resulting nanofiber membrane exhibits robust hydrophobicity alongside high solar reflectance (91.7%) and thermal emissivity (96.9%). This combination enables a 6.9 °C sub‐ambient temperature drop, making it ideal for energy‐saving, water‐resistant textiles and wearable technologies.

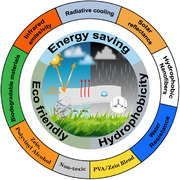

## 1 Introduction

Given the escalating global warming crisis,^[^ ^1^, ^2^, ^3^, ^4^ ^]^ the development of innovative and sustainable cooling technologies has become imperative. Among conventional approaches, radiative cooling (RC) offers a highly promising passive solution by dissipating heat into outer space by emitting thermal radiation through the atmospheric transparency window (8–13 µm).^[^ ^5^, ^6^, ^7^ ^]^ Unlike energy‐intensive conventional cooling systems, RC operates without external power, offering an environmentally friendly approach to address the burgeoning global cooling demands.^[^ ^5^, ^8^, ^9^, ^10^ ^]^ The increasing urgency of climate change and the global energy crisis have intensified research focused on developing scalable, durable, and sustainable RC materials.^[^ ^11^, ^12^, ^13^, ^14^, ^15^, ^16^, ^17^ ^]^

RC materials achieve subambient cooling by combining high reflectance across the solar spectrum (0.2–2.5 µm) with high emissivity in the long‐wave infrared (LWIR) range (8–13 µm).^[^ ^5^, ^7^, ^8^, ^9^, ^10^, ^18^, ^19^, ^20^ ^]^ This dual functionality enables RC materials to cool surfaces passively below ambient temperatures, even under direct sunlight. Polyvinyl alcohol (PVA), owing to its high LWIR emissivity, mechanical strength, and flexibility, serves as a distinctive candidate for RC composites, offering both structural integrity and versatility for diverse RC applications.^[^ ^21^, ^22^, ^23^ ^]^ Enhancing the potential of PVA‐based RC composites involves the integration of silicon dioxide (SiO_2_) and aluminum oxide (Al_2_O_3_) nanoparticles.^[^ ^14^, ^23^, ^24^, ^25^, ^26^, ^27^ ^]^ Specifically, SiO_2_ nanoparticles significantly increase solar reflectance via efficient scattering,^[^ ^28^, ^29^ ^]^ thereby minimizing solar heat gain in PVA composites. In addition, SiO_2_ exhibits high emissivity in the LWIR range, centered at 9 µm, owing to the vibrational modes of the Si─O─Si bonds.^[^ ^22^, ^23^, ^30^, ^31^, ^32^, ^33^ ^]^ Similarly, Al_2_O_3_ nanoparticles enhance radiative heat dissipation by maximizing LWIR emissivity^[^ ^14^, ^34^, ^35^ ^]^ and contribute to solar reflection via Mie scattering. The combined use of SiO_2_ and Al_2_O_3_ nanoparticles produces complementary emissivity profiles, enhancing LWIR radiation across a broader range within the atmospheric window. Specifically, within the atmospheric window of 8–13 µm, SiO_2_ peaks at ≈9 µm,^[^ ^22^, ^36^, ^37^, ^38^ ^]^ and Al_2_O_3_ exhibits strong emissivity between 10 and 13 µm,^[^ ^14^, ^25^, ^26^, ^27^, ^35^, ^37^, ^38^ ^]^ resulting in efficient and spectrally extended heat dissipation. Despite these advantages, the intrinsic hydrophilicity of PVA,^[^ ^39^, ^40^ ^]^ reflected in a low water contact angle of ≈40°,^[^ ^41^ ^]^ significantly reduces material durability, particularly under humid conditions. This moisture sensitivity remains a critical limitation for the broader implementation of PVA‐based RC composites in real‐world environments.^[^ ^42^, ^43^ ^]^

**Figure 1:**
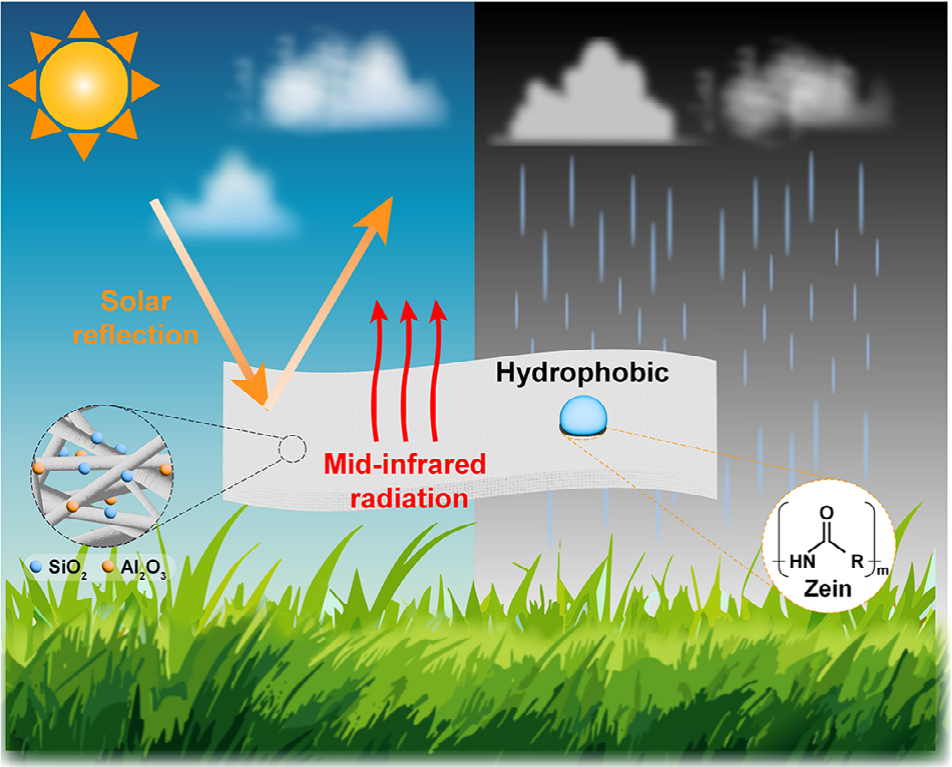
PZAS nanofiber membrane, which exhibits enhanced radiative cooling performance through solar reflection and mid‐infrared emission, with water resistance resulting from its hydrophobic properties.

Efforts to address the hydrophilicity of PVA, while preserving its advantageous properties, remain critical for expanding its applicability. However, conventional strategies to enhance PVA's water resistance, such as chemical crosslinking, often involve toxic reagents or complex processes.^[^ ^44^, ^45^, ^46^, ^47^, ^48^, ^49^, ^50^ ^]^ While inherently hydrophobic polymers such as PVDF are an alternative, they involve significant drawbacks, including high production cost, the use of hazardous solvents like NMP,^[^ ^51^ ^]^ and persistent environmental pollutants such as PFAS.^[^ ^51^, ^52^, ^53^ ^]^ These limitations highlight the need for a safe, scalable, and non‐toxic strategy to advance RC technologies based on PVA. Zein, a hydrophobic protein derived from corn, presents a potential solution to the aforementioned limitations of PVA. Its inherent hydrophobicity enhances the moisture resistance of PVA nanofibers via hydrophobic interactions.^[^ ^54^ ^]^ Furthermore, Zein is non‐toxic, biodegradable, and derived from renewable resources^[^ ^55^ ^]^ and can be uniformly blended with PVA.^[^ ^56^, ^57^ ^]^ The use of Zein to address the inherent hydrophilicity of PVA in the context of RC composites has not been previously reported. In this study, we developed a composite nanofiber membrane, PVA/Zein/Al_2_O_3_/SiO_2_ (PZAS) via electrospinning and demonstrated its RC effect (**Figure** **1**). The optimized composition, PZAS‐10:2 (comprising 10 wt.% Al_2_O_3_ and 2 wt.% SiO_2_), exhibited a high average solar reflectance of 91.7% and strong LWIR emissivity of 96.9%, achieving up to 6.9 °C sub‐ambient cooling under direct sunlight. Additionally, the membrane demonstrated enhanced hydrophobicity, with an average water contact angle of 118.5° observed over 60 s, thereby overcoming the intrinsic moisture sensitivity of PVA without the use of toxic solvents. Such performance attributes, combined with superior optical properties, environmental stability, and a scalable fabrication method, position a PZAS membrane as a viable, eco‐friendly, high‐performance solution for next‐generation RC technologies.

## 2 Results and Discussion

### 2.1 Morphology and Structural Characteristics

The material's structure was investigated by Fourier transform infrared (FTIR) and X‐ray diffraction (XRD) analyses (Figure S1, Supporting Information). The XRD pattern showed broad peaks ≈8.9° and 19.5° 2θ, corresponding to the amorphous halo of Zein and the semi‐crystalline peak of PVA, respectively.^[^ ^58^, ^59^, ^60^, ^61^ ^]^ Sharp diffraction peaks from crystalline Al_2_O_3_^[^ ^62^ ^]^ and a broad hump from amorphous SiO_2_ were also observed, confirming the integration of all components. Furthermore, the FTIR spectrum showed the characteristic peaks of Zein (Amide I & II)^[^ ^58^, ^60^ ^]^ and a shift in the O─H band of PVA, indicating the incorporation of both polymers and the formation of hydrogen bonds.^[^ ^58^, ^61^, ^63^ ^]^ To determine how these structural features translate into mechanical performance, tensile tests were conducted. The resulting stress–strain profile (Figure S2, Supporting Information) indicates the PZAS nanofiber membrane is tough and ductile, showing reduced brittleness compared to pure zein fibers.^[^ ^60^ ^]^

**Figure 2:**
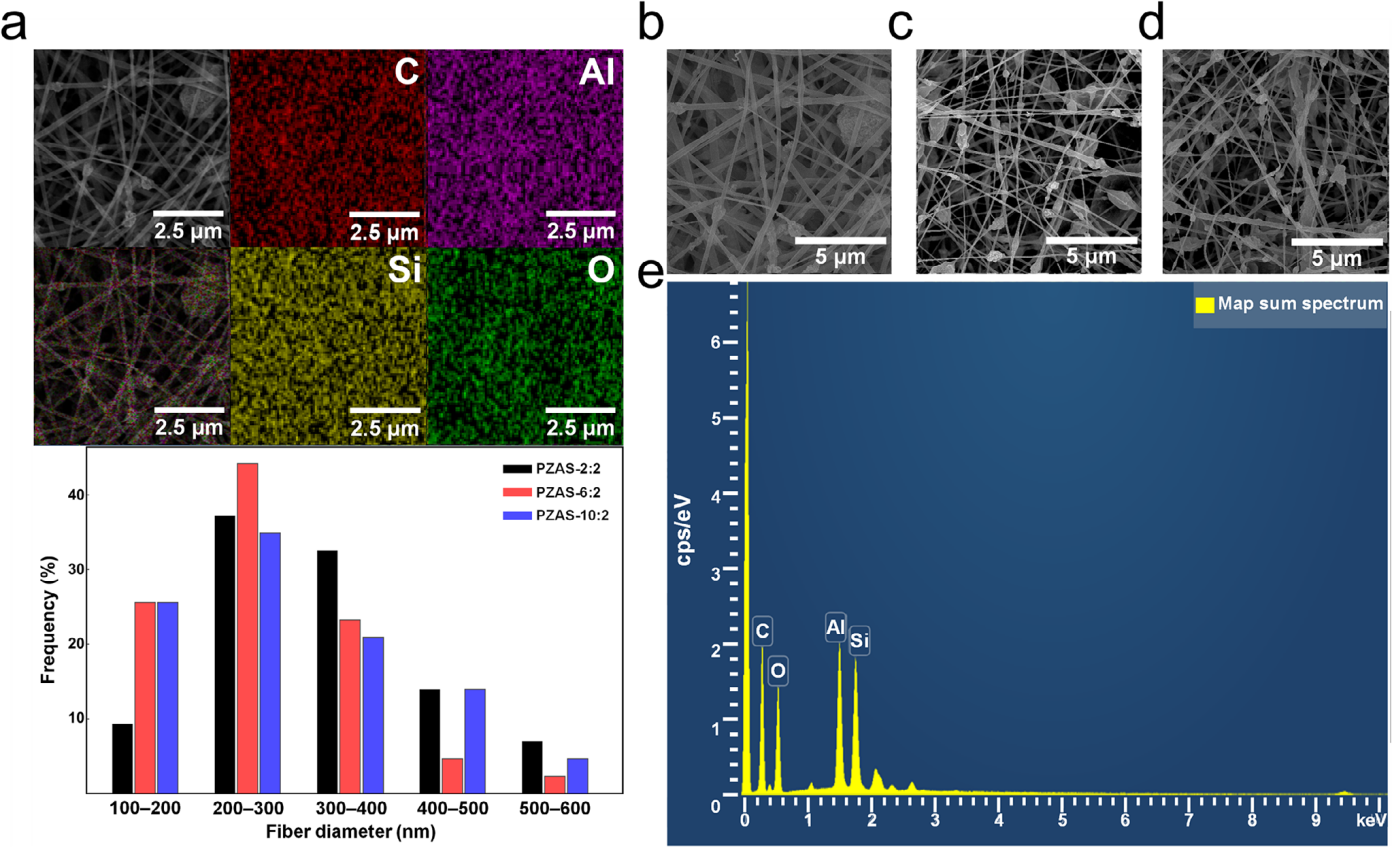
a) EDS elemental mapping of PZAS‐2:2 and diameter distributions of PZAS‐2:2, PZAS‐6:2, and PZAS‐10:2. b) SEM images of PZAS‐2:2, c) PZAS‐6:2, and d) PZAS‐10:2 e) Map sum spectrum of PZAS‐2:2.

**Figure** **2** illustrates the morphology, elemental composition, and diameter distribution of the electrospun PZAS nanofiber membranes. The elemental analysis by EDS, including the mapping (Figure 2a) and sum spectrum (Figure 2e), confirms the uniform dispersion of silicon and aluminum throughout the nanofiber membrane, indicating a homogeneous distribution of the incorporated Al_2_O_3_ and SiO_2_ nanoparticles. The SEM images of the PZAS‐2:2, PZAS‐6:2, and PZAS‐10:2 membranes (Figure 2b–d) reveal a consistent nanofiber distribution forming an interconnected porous network across all samples.

This multi‐scale structure is responsible for the material's high solar reflectance. This high reflectance is attributed to Mie scattering.^[^ ^64^, ^65^, ^66^ ^]^ The broad nanofiber diameter distribution (100–599 nm) (Figure 2a), which is comparable to solar wavelengths, promotes broadband scattering, while the interconnected porous network further enhances reflectivity through multiple scattering.

### 2.2 Hydrophobicity and Water Resistance

The enhanced hydrophobicity of the PZAS nanofibers is attributed to the blending of the hydrophobic Zein protein, which lowers the composite's surface energy.^[^ ^67^ ^]^ This composite is stabilized by intermolecular hydrogen bonding with the PVA matrix, as confirmed by a distinct shift in the O─H stretching band in the FTIR spectrum (Figure S1, Supporting Information). This approach significantly mitigates the inherent water sensitivity of PVA in radiative cooling membranes.

**Figure 3:**
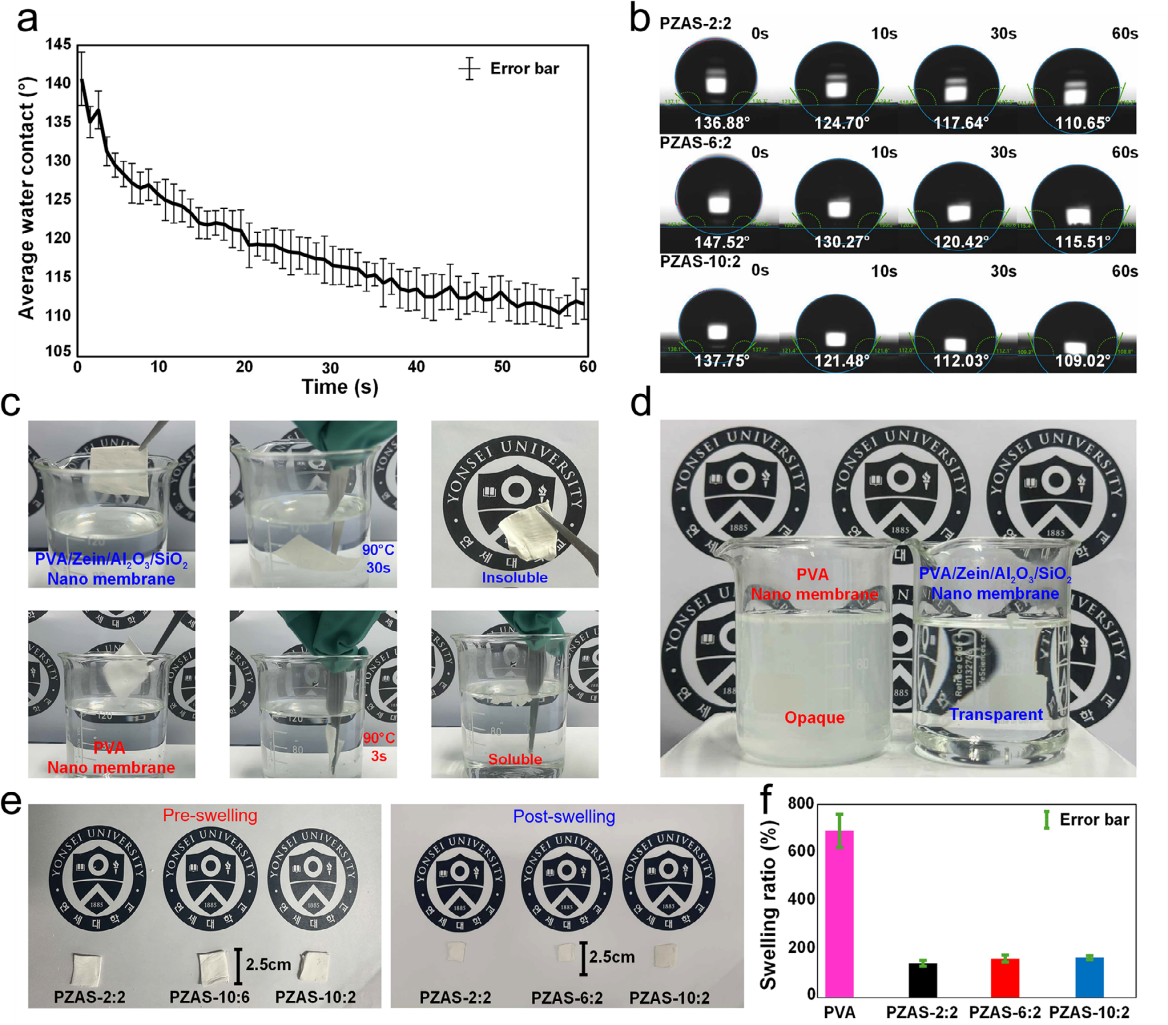
a) Average water contact angles of PZAS‐2:2, PZAS‐6:2, and PZAS‐10:2 membranes over 60 s. Each data point represents the average of three different compositions (PZAS‐2:2, PZAS‐6:2, and PZAS‐10:2), and the error bars indicate the standard error (S.E.) of the mean. b) Water contact image of PZAS. c) Hot water immersion test results for nanofiber membranes. d) Transparency of water after membrane immersion. e) Initial state of PZAS prior to the water swelling test and swollen state of PZAS after 24 h water immersion. f) Swelling ratios of PZAS and PVA nanofiber membranes. Data are presented as the mean ± S.E. (*n* = 6).

The static water contact angle measurements reveal an initial contact angle of ≈140°, indicating near‐superhydrophobicity (**Figure** **3**a,b). Over the 60 s measurement period, the average contact angle is 118.5°, with values decreasing from this initial value to ≈110° at the end. This decrease over time indicates sustained hydrophobicity with gradual water absorption (Figure 3a,b; Videos S1–S3, Supporting Information). All PZAS samples demonstrated functionally equivalent hydrophobicity, with high initial water contact angles (>136°) converging to a similar final value (∼112°± 4°). The minor variations in the contact angle profile are attributed to morphological differences inherent to the electrospinning process, and no significant reduction in hydrophobicity was observed with increasing Al_2_O_3_ content.

The hot water immersion test results demonstrate the superior structural water resistance of the PZAS nanofiber membrane compared to the PVA nanofiber membrane. Whereas the PVA nanofiber membrane dissolves within 3 s in 90 °C water, the PZAS nanofiber membrane maintains its structural integrity for at least 30 s (Figure 3c). The visual confirmation of this contrast in Figure 3d, illustrating opaque water with dissolved PVA and transparent water with PZAS, establishes the enhanced durability of the PZAS membrane in aqueous environments.

To confirm that the Zein modification overcomes the inherent water sensitivity of PVA, a hot water immersion test was conducted. This test provides a clear visual demonstration of the PZAS membrane's structural integrity under harsh aqueous conditions (90°C water), where an unmodified PVA control rapidly dissolves. The swelling ratio measurements in Figure 3f further elucidate the resistance of the PZAS composite membranes to water uptake and swelling. The average swelling ratios for PZAS‐2:2, PZAS‐6:2, and PZAS‐10:2 are 141.59 ± 12.20%, 161.57 ± 14.48%, and 165.73 ± 7.48%, respectively. In stark contrast, the PVA nanofiber membranes exhibit a significantly higher average swelling ratio of 690.14 ± 69%. These results confirm the superior resistance of the PZAS membranes to water absorption and volumetric expansion. The minimal swelling observed in the PZAS membranes, even alongside the gradual water absorption indicated by the decreasing contact angle over time, underscores their enhanced durability and suitability for performance in humid or aqueous environments. This controlled interaction with water, absorbing gradually without significant structural expansion, is a key characteristic for maintaining stability in challenging environments (Figure 3e).

Our approach imparts significant water resistance (Contact Angle >136°) to the PVA matrix through a simple, single‐step, and scalable process. This methodology distinguishes our work from conventional modifications that often require multi‐step syntheses or stringent reaction conditions, as detailed in the comparative analysis in Table S1 (Supporting Information).

### 2.3 Optical Properties and Radiative Cooling Performance

**Figure 4:**
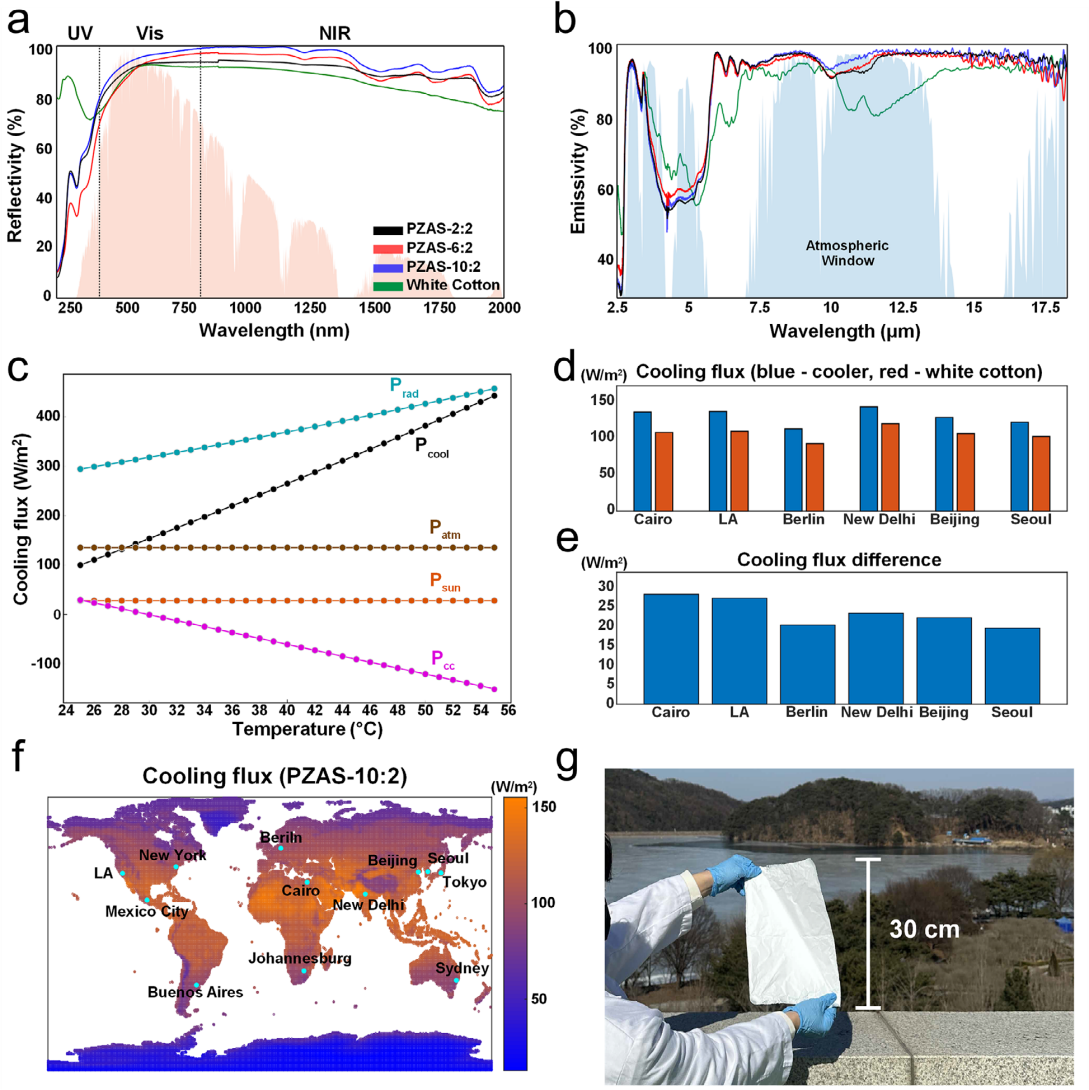
a) Solar reflectivity spectra of PZAS (pink area: normalized solar irradiance spectrum). b) LWIR emissivity spectra of PZAS (blue area: atmospheric transmittance). c) Cooling flux versus temperature for PZAS‐10:2. d) Cooling flux of PZAS‐10:2 and white cotton. e) Cooling flux difference between PZAS‐10:2 and white cotton in major cities. f) Global cooling flux map and g) sample image of PZAS‐10:2.

In this study, three PZAS nanofiber membranes were synthesized by varying the Al_2_O_3_ concentration while maintaining a fixed SiO_2_ content of 2 wt.%.^[^ ^22^ ^]^ The PZAS nanofiber membranes demonstrate outstanding performance in both solar reflectivity and LWIR emissivity. The Mie scattering results from the UV to NIR region for the PZAS nanofiber membrane series exhibited high solar reflectivity, starting at 87.7% for PZAS‐2:2 and reaching 91.7% for the optimized PZAS‐10:2 membrane (**Figure** **4**a). Concurrently, the LWIR emissivity remained high: 95.3% for PZAS‐2:2 and increasing to 96.9% for PZAS‐10:2 (Figure 4b). This enhanced emissivity is attributed to the fact that SiO_2_ displays an emissivity peak at ≈9 µm,^[^ ^22^, ^36^, ^37^, ^38^ ^]^ whereas Al_2_O_3_ has emissivity peaks within the 10–13 µm range.^[^ ^14^, ^25^, ^26^, ^27^, ^35^, ^37^, ^38^ ^]^ These non‐overlapping peaks contribute to broad and strong emissivity across the entire atmospheric transparency window, facilitating efficient thermal emission. Considering both the high solar reflectivity and LWIR emissivity, the PZAS nanofiber membranes effectively provide superior optical properties for radiative cooling compared with white cotton (solar reflectivity of 83.6% and LWIR emissivity of 89.29%).

The simulated cooling performance of the PZAS nanofiber membrane was analyzed using ViBA Rad software,^[^ ^68^ ^]^ and the results are presented in Figure 4c–f. Figure 4c presents the calculated cooling flux components for the PZAS‐10:2 membrane as a function of surface temperature. The membrane's radiative heat flux (P_rad_) increases with temperature, consistent with the Stefan–Boltzmann law. Under the calculated conditions, the absorbed solar (P_sun_) and atmospheric (P_atm_) radiation terms are fixed. The non‐radiative heat gain from convection and conduction (P_cc_) increases as the membrane surface cools relative to the ambient air, owing to the larger temperature gradient driving heat transfer. The net cooling flux (P_cool_), determined from Equation (6), remains positive over the entire temperature range examined, confirming the membrane's ability to sustain passive, sub‐ambient cooling under these calculated conditions. This finding indicates that spontaneous cooling occurs within the operational range without requiring external power consumption. The cooling performance was further characterized by comparing major cities (Figure 4d) and assessing the cooling flux difference (Figure 4e). Figure 4d demonstrates the higher cooling effect of PZAS compared with that of white cotton, across six major cities. The difference in cooling flux between PZAS and white cotton is depicted in Figure 4e, which shows positive values for all the cities analyzed. The global cooling flux distribution is presented in Figure 4f as part of these comprehensive simulation results.

### 2.4 Outdoor Cooling Performance

**Figure 5:**
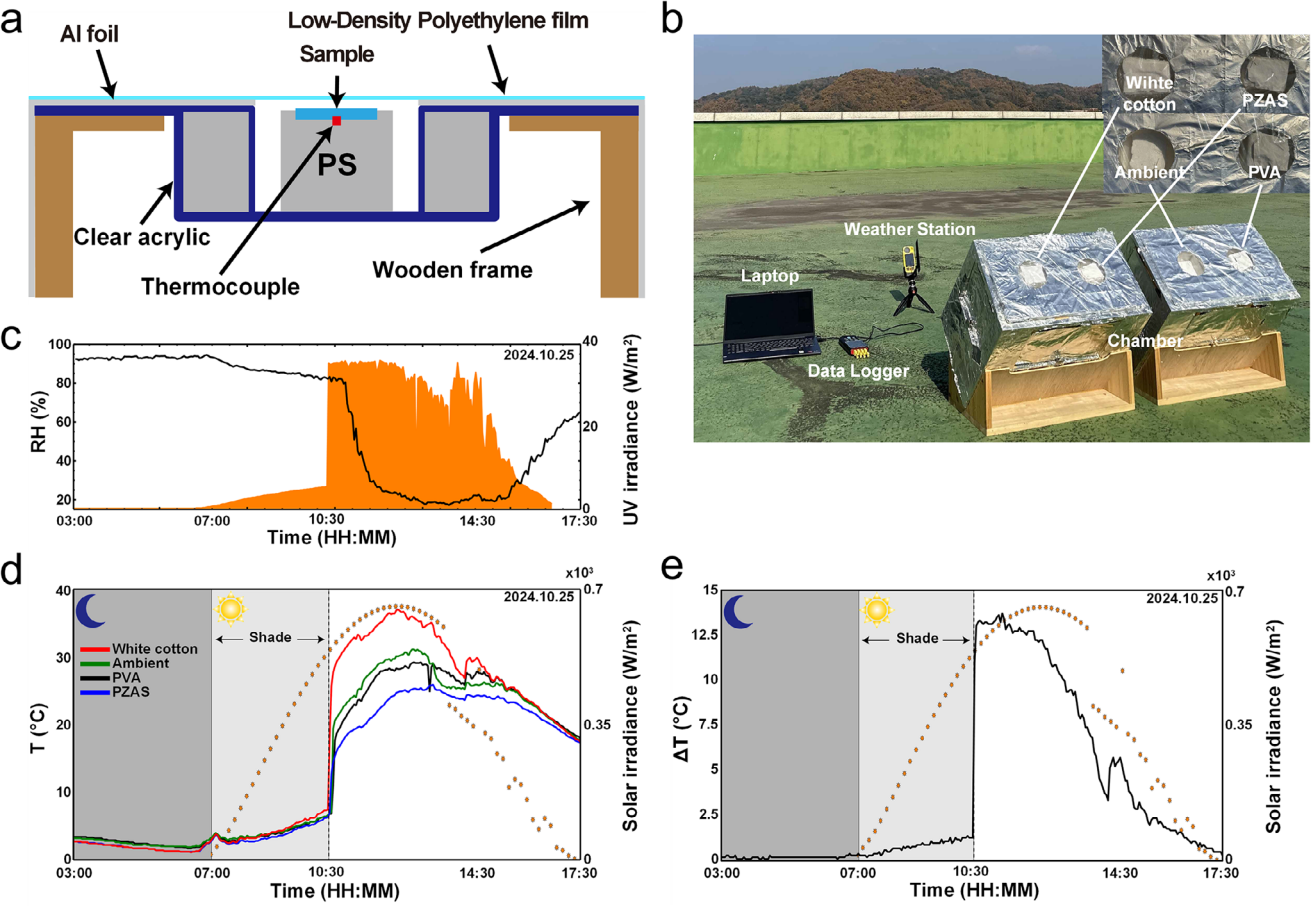
Outdoor temperature measurement setup and results. a) Schematic diagram and b) image of the setup. c) Temporal variations in on‐site relative humidity and UV irradiance, measured by a local weather station for environmental monitoring. d) Temporal variations in the surface temperatures of the samples. The secondary y‐axis shows the total solar irradiance data provided by the Korea Institute of Energy Research (KIER) for quantitative performance analysis. e) Temperature difference between white cotton and the PZAS‐10:2 membrane, correlated with the KIER solar irradiance data.

Outdoor temperature measurements were conducted to evaluate the cooling performance of the PZAS nanofiber membranes (**Figure** **5**a,b). The relative humidity and UV irradiance were monitored using a weather station (Figure 5c). The west‐facing orientation of the outdoor measurement setup resulted in a substantial increase in direct solar irradiance commencing at ≈10:30 AM, which corresponded to the sharp peaks observed in both the UV irradiance and temperature measurements. The surface temperatures of the PZAS‐10:2 membrane, PVA nanofiber membrane, and white cotton samples were measured and are shown in Figure 5d. Throughout the measurement period, during which the average ambient temperature was 14.08 °C, the PZAS‐10:2 membrane and white cotton exhibited average surface temperatures of 12.33 and 15.79 °C, respectively. Notably, during the peak solar irradiance period (10:30 AM–2:30 PM), the average surface temperatures recorded were 32.98 °C for white cotton, 22.95 °C for PZAS‐10:2, 27.23 °C for ambient, and 26.50 °C for the PVA nanofiber membrane. During this period, the PZAS‐10:2 membrane remained on average 3.55 °C lower than the PVA nanofiber membrane (Figure 5d). Moreover, the PZAS‐10:2 membrane achieved a maximum temperature reduction of 6.9 °C below ambient and demonstrated a 13.7 °C lower temperature compared with white cotton (Figure 5d,e). Although white cotton exhibits high solar reflectivity, the PZAS‐10:2 nanofiber membrane is higher (Figure 4a). Furthermore, the inclusion of Al_2_O_3_ and SiO_2_ nanoparticles within the PZAS‐10:2 membrane enhances both the solar reflectivity and LWIR emissivity compared with the bare PVA. This spectral enhancement results in lower temperatures compared with those of the normal PVA nanofiber membrane.

To assess the material's stability under humid conditions, we conducted an additional outdoor cooling test on a high‐humidity day (>75% RH), where the PZAS membrane consistently maintained a lower surface temperature than an unmodified PVA control (Figure S3a, Supporting Information). Optical analysis of a swelled membrane revealed that while solar reflectance decreases upon wetting, the LWIR range emissivity changed by less than 1.5% (Figure S3b, Supporting Information), thereby preserving its cooling function.

## 3 Conclusion

This work demonstrates a simple, non‐toxic, and scalable approach to impart stable hydrophobicity to PVA‐based radiative cooling membranes. The incorporation of biocompatible Zein addresses the moisture sensitivity limitations of PVA by achieving high initial hydrophobicity with a transition to gradual water absorption, thereby ensuring material suitability for human‐centric and direct‐contact applications. We introduced a scalable and eco‐conscious RC technology based on this unique PZAS, which achieves optimal solar reflectivity and efficient thermal dissipation through its novel design. The distinctive wettability characteristics of PZAS further broaden its potential in advanced biomedical applications such as moisture‐managing textiles, sweat‐based biosensors, and controlled drug delivery systems. The PZAS nanofiber membrane, produced via a scalable electrospinning method, was validated through real‐world outdoor experiments that confirmed its superior cooling performance and practical applicability. This material demonstrates substantial promise for widespread and cost‐effective deployment across diverse sectors.

## 4 Experimental Section

### Materials

PVA (1799, Aladdin Biochemical Technology Co., Ltd., China) and Zein (Sigma–Aldrich, USA) were used as the polymer matrix. Al_2_O_3_ (80 nm, US Research Nanomaterials, USA) and SiO_2_ (20–30 nm, Avention, Korea) nanoparticles were incorporated into the composite to enhance solar reflectance across the solar spectrum and to increase emissivity within the atmospheric window. Deionized (DI) water and glacial acetic acid (99.7%, Samchun, Korea) were used as solvents.

### Preparation of PZAS Nanofiber Membrane Solutions

**Figure 6:**
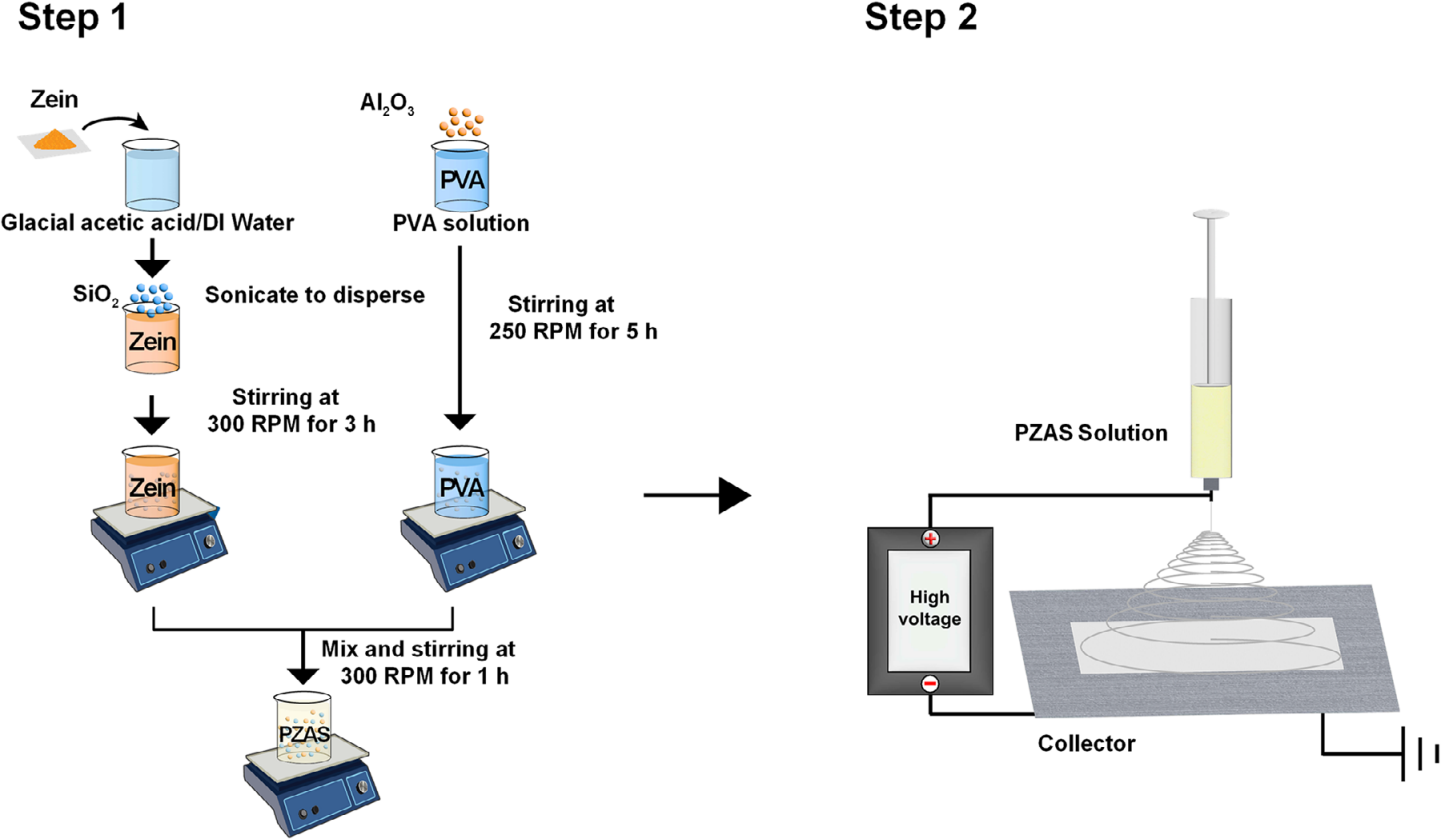
Schematic of the fabrication process of PZAS nanofiber membranes.

PZAS nanofiber solutions were prepared with varying Al_2_O_3_ and SiO_2_ nanoparticle concentrations, as illustrated in **Figure** **6**. A 10 wt.% PVA solution was prepared by dissolving PVA in DI water at 98 °C for 5 h. Subsequently, Al_2_O_3_ nanoparticles were added to the PVA solution to achieve final concentrations of 2, 6, and 10 wt.% relative to the total solution weight, followed by stirring at 50 °C to ensure homogeneous dispersion. Concurrently, a 10 wt.% Zein solution was prepared by dissolving Zein in an 8:2 v/v mixture of glacial acetic acid and DI water at room temperature for 3 h. SiO_2_ nanoparticles were subsequently added to the Zein solution to a final concentration of 2 wt.% relative to the total solution weight, followed by ultrasonication for 2 h and an additional 3 h of stirring. Finally, the PVA/Al_2_O_3_ and Zein/SiO_2_ solutions were combined in a 2:3 volume ratio and mixed for 1 h. This process resulted in three distinct PZAS solutions with final Al_2_O_3_ and SiO_2_ concentrations of 2:2, 6:2, and 10:2 wt.% (denoted as PZAS‐2:2, PZAS‐6:2, and PZAS‐10:2, respectively).

### Preparation of PVA Nanofiber Membrane

For comparative analysis, a pure PVA nanofiber membrane was fabricated as a control sample. A 10 wt.% PVA solution was prepared by dissolving PVA powder in DI water and stirring at 98 °C for 5 h. The solution was electrospun at an applied voltage of 19 kV, a flow rate of 0.8 mL h^−1^, and a spinneret‐to‐collector distance of 15 cm. The collected membrane was dried in a vacuum oven at 100 °C for 3 h to remove any residual solvent.

### Preparation of Zein Nanofiber Membrane

For FTIR analysis, a pure Zein nanofiber membrane was fabricated as a control sample. A 30 wt.% Zein solution was prepared by dissolving Zein powder in an 8:2 (v/v) mixture of glacial acetic acid and DI water and stirring at room temperature for 5 h. The solution was electrospun at an applied voltage of 24 kV, a flow rate of 0.6 mL h^−1^, and a spinneret‐to‐collector distance of 15 cm. The collected membrane was dried in a vacuum oven at 80 °C for 2 h to remove any residual solvent.

### Electrospinning of PZAS Nanofiber Membranes

PZAS nanofiber membranes were fabricated via electrospinning. The prepared PZAS solutions (4 mL total) were loaded into a syringe pump and electrospun under the following optimized conditions: an applied voltage of 24.5 kV, a flow rate of 1 mL h^−1^, and a spinneret‐to‐collector distance of 12 cm. The process was conducted at a relative humidity of ≈35%. The nanofibers were collected on an aluminum foil substrate. Residual solvents were removed by drying the membranes in a vacuum oven at 100 °C for 3 h. The membrane thickness was controlled by adjusting the electrospinning duration.

### Optical and Radiative Cooling Performance–Spectral Reflectance and Emissivity Measurements

The optical properties of the PZAS nanofiber membranes were characterized as follows. The solar reflectance was measured in the 0.2–2.0 µm wavelength range employing UV–vis–near‐infrared (NIR) spectrophotometry (Jasco 770, Jasco, Japan). The change in reflectance of the PZAS samples upon swelling was measured in the 0.5–2 µm wavelength range by a UV–vis–NIR spectrophotometer (LAMBDA 950, Perkin Elmer, USA). The LWIR emissivity in the 8–13 µm atmospheric transparency window was determined via diffuse reflectance measurements employing Fourier transform infrared spectroscopy (Vertex 70v, Bruker, Germany).

The emissivity (*ε*) was calculated according to Kirchhoff's law:

(1) ε=1−ρ−t

where *ɛ* is the emissivity, *ρ* is the reflectivity, and *t* is the transmissivity.

### Radiative Cooling Performance Simulation

To comprehensively evaluate the cooling performance, the proposed PZAS nanofiber membrane was analyzed utilizing ViBA Rad,^[^ ^68^ ^]^ an open‐source software designed for assessing RC materials. This analysis included the cooling flux of the PZAS membrane and a global cooling flux map. The following equations were employed to determine the RC flux components, i.e., the radiative heat flux emitted from the membrane (*P_rad_*), atmospheric radiation absorbed by the membrane (*P_atm_*), solar radiation absorbed by the membrane (*P_sun_*), and heat loss via convection and conduction (*P_cc_*):

(2) $$P_{rad}=2\pi \int \;\;\;\int \sin\theta \;cos\theta \;I_{BB}\left(T,\lambda \right)\varepsilon \left(\lambda ,\theta \right)d\lambda d\theta$$

(3) $$P_{atm}=2\pi \int \;\;\;\int \sin\theta \;cos\theta \;I_{BB}\left(T_{amb},\lambda \right)\varepsilon_{amb}\left(\lambda \right)\varepsilon \left(\lambda ,\;\theta \right)d\lambda d\theta$$

(4) $$P_{sun}=\int I_{AM}\left(\lambda \right)\varepsilon \left(\lambda ,\theta_{sun}\right)d\lambda$$

(5) $$P_{cc}=h_{cc}\left(T_{amb}-T\right)$$

where *θ* is the incident angle (*θ_sun_* for solar incident angle), *T* is the PZAS membrane surface temperature, *T_amb_* is the ambient temperature, and *ɛ*(*λ*, *θ*) is the emissivity of the PZAS membrane. The emissivity of the ambient atmosphere, ɛ_*amb*_(λ), is given by ɛ_*amb*_ (λ) =  1 − *t*(λ)^1/cosθ^, where *t*(*λ*) represents the atmospheric transmittance spectrum. *I_BB_* denotes the spectral irradiance of a blackbody at temperature *T*, and *I_AM_* (*λ*) is the solar spectral irradiance. The net cooling flux (*P_cool_*) was then calculated using:

(6) $$P_{cool}=P_{rad}-\left(P_{sun}+P_{atm}+P_{cc}\right)$$

To analyze the cooling performance, the spectral reflectance and emissivity of the PZAS nanofiber membranes were first measured and these spectra were imported into the open‐source software ViBA Rad. After setting the model and environmental parameters described below, the software calculated the net cooling flux (*P_cool_*) according to Equations (2)–(6). In these calculations, the PZAS membrane temperature ranged from 298 to 328 K, the ambient temperature (*T_amb_*) was set to 303 K, and the combined heat transfer coefficient (*h_cc_*) was fixed at 8 W m^−^^2^ K. For the external environmental conditions, the standard AM1.5 Global solar spectrum was used for solar irradiance (*I_AM_*), and the atmospheric transmittance spectrum for AM1.5 with 1 mm of precipitable water was used for atmospheric radiation calculations.

### Outdoor Radiative Cooling Performance Evaluation

The experimental setup was designed to evaluate the RC performance of samples under direct solar exposure. The samples were positioned to face the west sky, maximizing their exposure to afternoon solar radiation. To mitigate convective heat transfer while allowing natural convection, a low‐density polyethylene film, transparent to both solar and infrared radiation, was placed over the sample container. The container itself was insulated with aluminum foil wrapped around a wooden frame, further enhanced by expanded polystyrene foam with air pockets to minimize solar absorption and conductive heat transfer. Temperature measurements were conducted utilizing K‐type thermocouples, selected for their reliability and accuracy across a wide temperature range. These measurements were continuously logged at 5 min intervals using a data logger (HH520, OMEGA engineering, USA), enabling a detailed analysis of the temperature fluctuations and dynamic cooling responses of the samples. For on‐site environmental monitoring, a local weather station was used to record the real‐time relative humidity and UV irradiance (Figure 5c). To accurately assess the cooling performance, total solar irradiance data were obtained from satellite measurements provided by the KIER and are plotted alongside the temperature data (Figure 5d,e).

### Morphology and Microstructure Analysis

The morphology and microstructure of the PZAS nanofiber membranes were characterized via scanning electron microscopy (SEM, Verios 5 UC, Thermo Fisher Scientific) and energy‐dispersive X‐ray spectroscopy (EDS). SEM imaging was performed to examine the uniformity of the nanofibers and surface roughness of the membranes. Fiber diameters were measured from SEM images (*n* = 43 per group for PZAS‐2:2, 6:2, and 10:2) using ImageJ software.^[^ ^69^ ^]^ EDS analysis was conducted to validate the elemental composition of the PZAS membranes.

### Mechanical Properties

The mechanical properties of PZAS‐10:2 nanofiber membranes were analyzed by tensile tests based on a modified ASTM D882 standard. The tests were conducted using a universal testing machine (UTM, Model 34SC‐1, Instron Corporation, USA). A tensile speed of 50 mm min^−1^ and an initial gauge length of 37 mm were used for all measurements. All tests were performed in triplicate for each condition, and the results are presented as the mean ± S.E. (*n* = 3).

### Chemical and Crystal Structure Analysis

A FTIR spectroscopy study was conducted to analyze the chemical structures of the nanofiber membranes using an FTIR spectrometer (Invenio, Bruker, USA), ranging from 4000 to 600 cm^−1^. Spectra were obtained directly from the nanofiber membrane surface using an attenuated total reflection (ATR) accessory. The crystal structure of the PZAS nanofiber membrane was analyzed by XRD (SmartLab, Rigaku, Japan). The patterns were recorded with Cu‐Kα radiation (λ = 1.5406 Å) operating at 45 kV and 200 mA. Data were collected over a 2θ range of 5°–80° at a scan speed of 2.5°/min.

### Water Resistance—Contact Angle Measurements and Swelling Test

The hydrophobicity of the PZAS nanofiber membranes was evaluated using static water contact angle measurements. The measurements were performed using the sessile drop method utilizing a contact angle goniometer (Model DSA100, KRUSS). A water droplet was dispensed onto the membrane surface, and the contact angle was measured. The swelling ratios of the PZAS‐2:2, PZAS‐6:2, and PZAS‐10:2 nanofibers, as well as the PVA nanofibers (control), were determined gravimetrically. Dried nanofiber samples (*W_d_*) were immersed in deionized water at room temperature for 24 h (*n* = 6 per group). Excess surface water was removed by blotting with Kimtech wipers for 20 s, and the wet weight (*W_w_*) was immediately measured. The swelling ratio (*S*) was calculated using the formula:

(7) $$S=\frac{W_{w}-W_{d}}{W_{d}}*100\%$$

## Conflict of Interest

The authors declare no conflict of interest.

## Supporting information



Supporting Information

Supplemental Video 1

Supplemental Video 2

Supplemental Video 3

## Data Availability

The data that support the findings of this study are available from the corresponding author upon reasonable request.
