# Supplementary material for: Hydrophobic Radiative Cooling using Zein‐Functionalized Polyvinyl Alcohol Nanofibers with Dielectric Nanoparticles
Source: Small. 2025 Sep 24;21(45):e07295. doi: 10.1002/smll.202507295 (PMC12614153; doi:10.1002/smll.202507295)
Supplement: Supplementary file 1 — Supporting Information [file SMLL-21-e07295-s002.docx]

Supporting Information

**Hydrophobic Radiative Cooling using Zein-Functionalized Polyvinyl Alcohol Nanofibers with Dielectric Nanoparticles**

*Minseo Jeong^1^, Seokgyu Kwon^1^, Changhwan Hyeon^1^, Juhoon Baek^2^, Myeongsu Seong^3^, Minkyung Kim^2 *^, Dasol Lee^1 *^*

^1^Department of Biomedical Engineering, Yonsei University, Wonju 26493, Republic of Korea

*E-mail: ([Dasol@yonsei.ac.kr](mailto:Dasol@yonsei.ac.kr))

^2^School of Mechanical and Robotics Engineering, Gwangju Institute of Science and Technology (GIST), Gwangju 61005, Republic of Korea

*E-mail: ([m.kim@gist.ac.kr](mailto:m.kim@gist.ac.kr))

^3^Department of Mechatronics and Robotics, School of Advanced Technology, Xi’an Jiaotong-Liverpool University, Suzhou 215123, China

| Modification Category | Specific Method/Reagent | Achieved WCA (°) | Summary of Process & Key Features |
| --- | --- | --- | --- |
| This work | Zein blending | >136°  (at t=0) | A sustainable, single-step process utilizing a natural corn protein (Zein) to impart hydrophobicity. |
| Chemical Crosslinking | Glutaraldehyde (GA)  [1] | Vapor Crosslinking  64.4° ± 2.3°  Solution Crosslinking  50.5° ± 2.7° | 1. Glutaraldehyde (GA) Vapor/Solution Treatment  2. Acid-Catalyzed Acetalization  3. Secured Structural Stability & Tunable Wettability |
|  | Citric Acid (CA)  [2] | < 42° | 1. Citric Acid (CA) Addition  2. Heat-Induced Esterification  3. Improved Water, Thermal & Mechanical Stability |
|  | Sulfosuccinic Acid (SSA)  [3] | 75° ~ 98° | 1. PVA/SSA Solution Casting  2. Heat-Induced Esterification  3. Improved Water Resistance (Reduced Swelling) & Increased Surface Hydrophobicity |
|  | Malic Acid  [4] | 87.8°  (After Malic Acid Crosslinking) | 1. Electrospinning CS/PVA with Malic Acid  2. Condensing Agent Treatment  3. Simultaneous Amide/Ester Crosslink Formation |
| Physical Modification | PCL Nanofiber Coating/Embedding  [5] | 58.9° ~ 119.6° | 1. Electrospinning Hydrophobic PCL Nanofibers  2. Physical Binding/Embedding onto Hydrophilic PVA Film Surface  3. Improved Water Resistance (Contact Angle), Mechanical & Oxygen Barrier Properties |
|  | LCNF-PEI blending  [6] | Max 104° | 1. Blending Lignin-Contained Nanofibers (LCNF-PEI) into PVA Solution  2. Solution Casting  3. Improved Water Resistance via Hydrophobicity (Lignin) & Increased Surface Roughness |
| Advanced Surface Treatment | Sol-Gel Silanization Coating after Heat Treatment  [7] | Max 144°  (After DTMS Sol-Gel Coating) | 1. Heat Treatment of PVA Nanofibers (Stabilization via Increased Crystallinity)  2. Coating with Silane Sol-Gel Solution  3. Formation of Hydrophobic Siloxane Surface Layer |
|  | Plasma Treatment (SF6)  [8] | Max 152.1° | 1. SF₆ Plasma Treatment on PVA Nanofibers  2. Surface Fluorination & Increased Microroughness  3. Formation of Superhydrophobic Surface |
|  | TiO₂-Silane Hybrid Coating  [9] | up to 157° | 1. Anchoring TiO₂ Nanoparticles on PVA Sponge Skeleton (Hierarchical Structure)  2. Coating with Low-Energy Material (FDTS)  3. Realization of Superhydrophobic Surface |
|  | PDMS/Hydrophobic SiO₂ Coating  [10] | 100° | 1. Immersion/Coating of PVA Nanofibers in PDMS/Hydrophobic SiO₂ Solution  2. Micro/Nano Structure Formation & Reduced Surface Energy  3. Realization of Hydrophobic Surface |

**Table S1. Comparative Analysis of Hydrophobic Modification Methods for PVA**


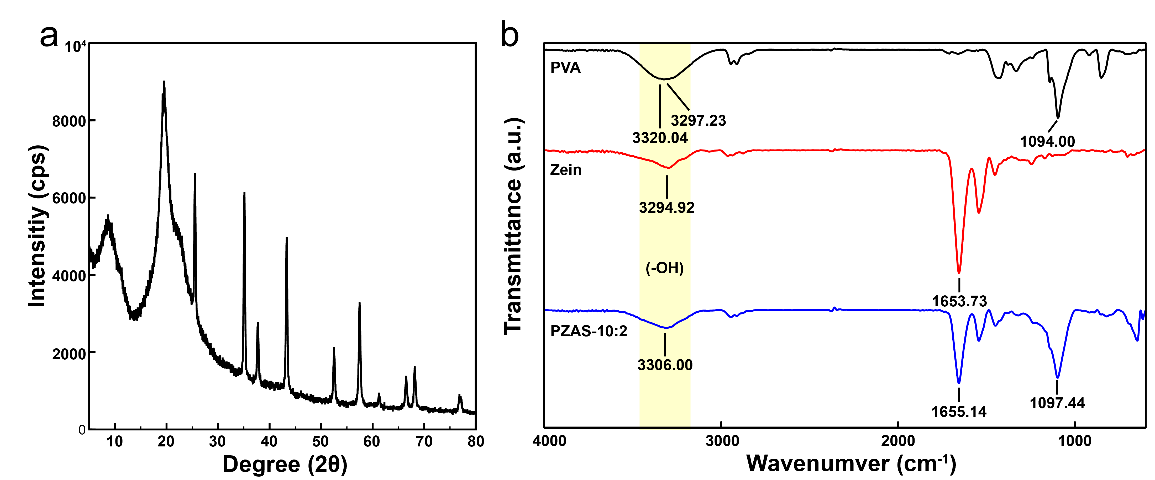
 **Figure S1.** (a) XRD pattern of PZAS-10:2 b) FTIR spectra of PVA, Zein, PZAS-10:2.

**
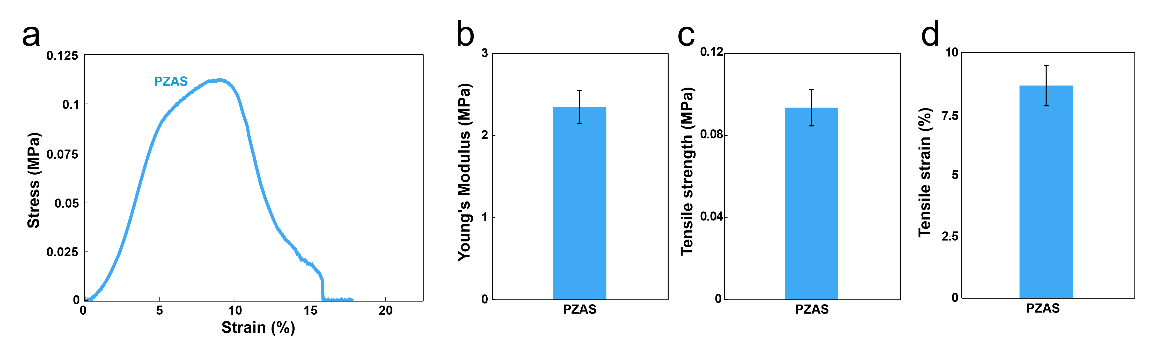
**

**Figure S2.** Mechanical properties of PZAS-10:2 nanofiber membranes. (a) Representative stress-strain curves. Comparison of (b) Young’s modulus, (c) tensile strength, and (d) tensile strain.

**
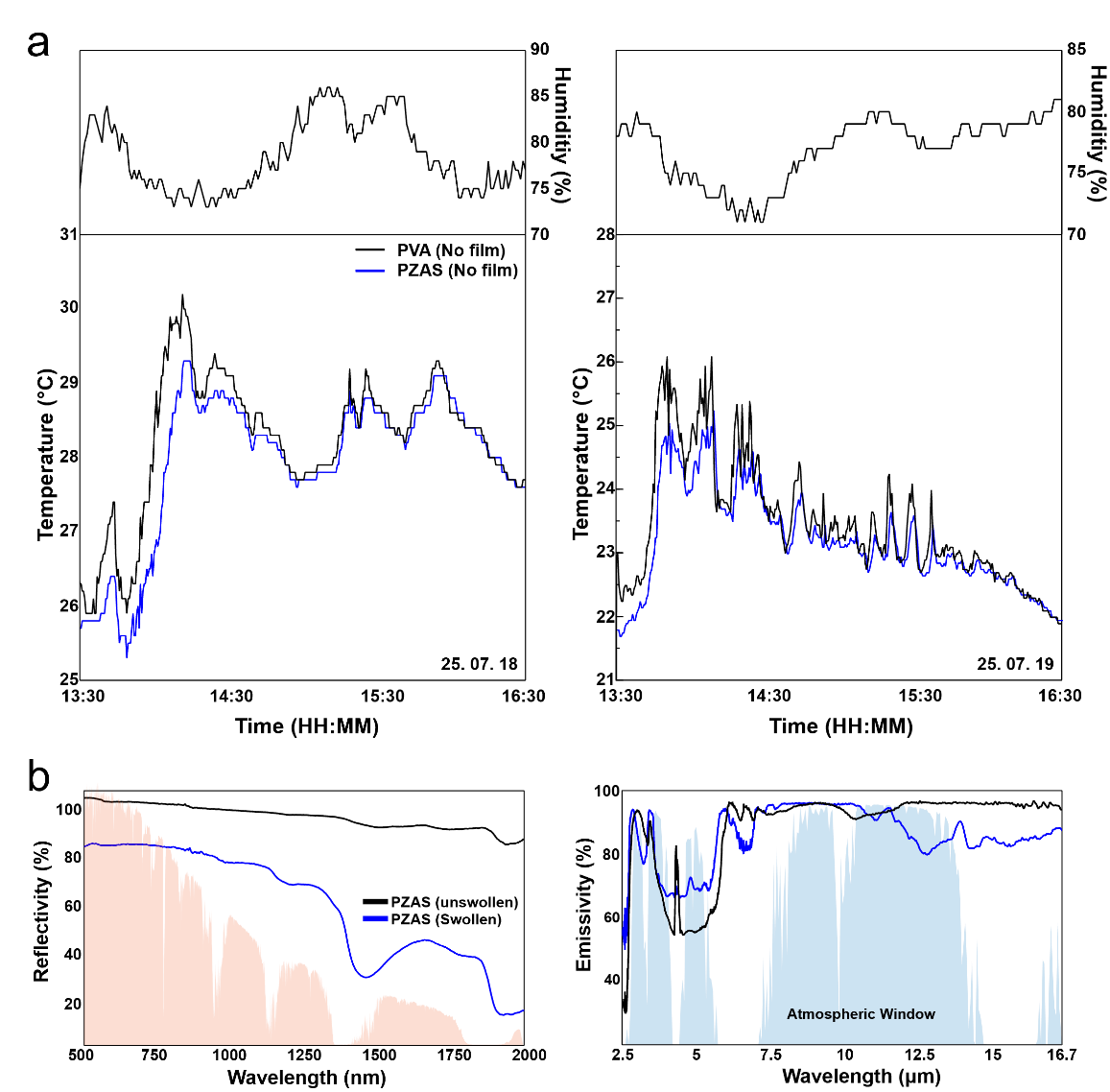
**

**Figure S3.** Outdoor performance and spectral analysis of the PZAS. (a) Outdoor temperature data of the PZAS sample measured at high relative humidity (The average humidity during the measurement period was 78.53 ± 3.82% standard deviation (SD) on July 18, 2025, and 76.95 ± 2.56% (SD) on July 19, 2025, the humidity data for this measurement was obtained from the Korea Meteorological Administration (KMA)). (b) Solar reflectivity and LWIR emissivity spectra of the PZAS sample before and after swelling (157.2%).

To confirm the integrity and suitability for this follow-up investigation, we first re-measured its dry-state emissivity spectrum in the 8–13 μm range and confirmed that it matched the original data. The results of this optical analysis on the swelled sample are presented in Figure S3b

**Supporting reference**

[1] S. Ullah, M. Hashmi, N. Hussain, A. Ullah, M. N. Sarwar, Y. Saito, S. H. Kim, I. S. Kim, “Stabilized nanofibers of polyvinyl alcohol (PVA) crosslinked by unique method for efficient removal of heavy metal ions,” Journal of Water Process Engineering 33 (2020): 101111. https://doi.org/10.1016/j.jwpe.2019.101111.

[2] D. Yu, Y.-Y. Feng, J.-X. Xu, B.-H. Kong, Q. Liu, H. Wang, “Fabrication, characterization, and antibacterial properties of citric acid crosslinked PVA electrospun microfibre mats for active food packaging,” Packaging Technology and Science 34 (2021): 361-370. https://doi.org/10.1002/pts.2566.

[3] E. Rynkowska, K. Fatyeyeva, S. Marais, J. Kujawa, W. Kujawski, “Chemically and Thermally Crosslinked PVA-Based Membranes: Effect on Swelling and Transport Behavior,” Polymers 11 (2019): 1799. <https://doi.org/10.3390/polym11111799>.

[4] J. Chen, S. Han, M. Huang, J. Li, M. Zhou, J. He, “Green crosslinked nanofibers membrane based on CS/PVA combined with polybasic organic acid for tympanic membrane repair,” International Journal of Polymeric Materials and Polymeric Biomaterials 71 (2022): 291-301. https://doi.org/10.1080/00914037.2020.1825084.

[5] K. Ahn, K. Park, K. Sadeghi, J. Seo, “New Surface Modification of Hydrophilic Polyvinyl Alcohol via Predrying and Electrospinning of Hydrophobic Polycaprolactone Nanofibers,” Foods 13 (2024): 1385. https://doi.org/10.3390/foods13091385.

[6] Y. Li, Y. Chen, Q. Wu, J. Huang, Y. Zhao, Q. Li, S. Wang, “Improved Hydrophobic, UV Barrier and Antibacterial Properties of Multifunctional PVA Nanocomposite Films Reinforced with Modified Lignin Contained Cellulose Nanofibers,” Polymers 14 (2022): 1705. https://doi.org/10.3390/polym14091705.

[7] P. Bhattacharyya, “Hydrophobic modification of water-borne poly(vinyl alcohol) electrospun nonwovens for advanced applications,” Polymers and Polymer Composites 30 (2022): 1-9. https://doi.org/10.1177/09673911221080526.

[8] A. Thongphud, B. Paosawatyanyong, P. Visal-athaphand, P. Supaphol, “Improvement of Hydrophobic Properties of the Electrospun PVA fabrics by SF₆ Plasma Treatment,” Advanced Materials Research 55-57 (2008): 625-628. https://doi.org/10.4028/www.scientific.net/AMR.55-57.625.

[9] Z. He, H. Wu, Z. Shi, Z. Kong, S. Ma, Y. Sun, X. Liu, “Facile Preparation of Robust Superhydrophobic/Superoleophilic TiO₂-Decorated Polyvinyl Alcohol Sponge for Efficient Oil/Water Separation,” ACS Omega 7 (2022): 7084-7095. https://doi.org/10.1021/acsomega.1c06775.

[10] Q. Zhang, J. Sun, X. Cao, H. Wei, R. Du, X. Liu, “Poly(vinyl alcohol) composite nanofiber membranes with hydrophobicity for daytime radiative cooling,” Composites Communications 48 (2024): 101947. https://doi.org/10.1016/j.coco.2024.101947.
